# Differences in the Thoracic Aorta by Region and Sex in a Murine Model of Marfan Syndrome

**DOI:** 10.3389/fphys.2017.00933

**Published:** 2017-11-15

**Authors:** Francesc Jiménez-Altayó, Anna-Maria Siegert, Fabio Bonorino, Thayna Meirelles, Laura Barberà, Ana P. Dantas, Elisabet Vila, Gustavo Egea

**Affiliations:** ^1^Departament de Farmacologia, de Terapèutica i de Toxicologia, Institut de Neurociències, Facultat de Medicina, Universitat Autònoma de Barcelona, Bellaterra, Spain; ^2^Departament de Biomedicina, Facultat de Medicina i Ciències de la Salut, Universitat de Barcelona, Barcelona, Spain; ^3^Institut d'Investigacions Biomèdiques August Pi i Sunyer, Barcelona, Spain; ^4^Institut Clínic del Tòrax, Institut d'Investigacions Biomèdiques August Pi i Sunyer, Barcelona, Spain

**Keywords:** Marfan syndrome, aortic aneurysm, sex differences, gender medicine, ascending thoracic aorta contraction, cyclooxygenase, nitric oxide synthase, elastin fragmentation

## Abstract

Marfan syndrome (MFS) is a hereditary disorder of the connective tissue that causes life-threatening aortic aneurysm, which initiates at the aortic root and can progress into the ascending portion. However, analysis of ascending aorta reactivity in animal models of MFS has remained elusive. Epidemiologic evidence suggests that although MFS is equally prevalent in men and women, men are at a higher risk of aortic complications than non-pregnant women. Nevertheless, there is no experimental evidence to support this hypothesis. The aim of this study was to explore whether there are regional and sex differences in the thoracic aorta function of mice heterozygous for the fibrillin 1 (*Fbn1*) allele encoding a missense mutation (*Fbn1*^C1039G/+^), the most common class of mutation in MFS. Ascending and descending thoracic aorta reactivity was evaluated by wire myography. Ascending aorta mRNA and protein levels, and elastic fiber integrity were assessed by qRT-PCR, Western blotting, and Verhoeff-Van Gieson histological staining, respectively. MFS differently altered reactivity in the ascending and descending thoracic aorta by either increasing or decreasing phenylephrine contractions, respectively. When mice were separated by sex, contractions to phenylephrine increased progressively from 3 to 6 months of age in MFS ascending aortas of males, whereas contractions in females were unchanged. Endothelium-dependent relaxation was unaltered in the MFS ascending aorta of either sex; an effect related to augmented endothelium-dependent hyperpolarization-type dilations. In MFS males, the non-selective cyclooxygenase (COX) inhibitor indomethacin prevented the MFS-induced enhancement of phenylephrine contractions linked to increased COX-2 expression. In MFS mice of both sexes, the non-selective nitric oxide synthase inhibitor L-NAME revealed negative feedback of nitric oxide on phenylephrine contractions, which was associated with upregulation of eNOS in females. Finally, MFS ascending aortas showed a greater number of elastic fiber breaks than the wild-types, and males exhibited more breaks than females. These results show regional and sex differences in *Fbn1*^C1039G/+^ mice thoracic aorta contractility and aortic media injuries. The presence of more pronounced aortic alterations in male mice provides experimental evidence to support that male MFS patients are at increased risk of suffering aortic complications.

## Introduction

Marfan syndrome (MFS) is an autosomal dominant hereditary disorder of connective tissue that results from mutations in the gene for fibrillin 1 (*Fbn1*), the major constitutive element of extracellular microfibrils (Dietz et al., 1991). Aortic complications, including severe aortic regurgitation or acute aortic dissection, are potentially life threatening (Murdoch et al., 1972). Prophylactic surgical repair of the ascending aorta has extended life in MFS over the past decades, but as a result, new features have emerged including aortic dilatation beyond the aortic root (Pyeritz, 2016). Although, both ascending and descending portions of thoracic aorta show mechanical abnormalities in MFS (Chung et al., 2007b; Bellini et al., 2016), aneurysmal expansion initiates at the aortic root and progresses into the ascending portion. This sequence of events could be at least partly associated with the more pronounced mechanical alterations of this aortic region (Bellini et al., 2016). Remarkably, exploration of the ascending portion of thoracic aorta reactivity in murine models of MFS has remained elusive. As far as we know, all vascular reactivity studies on aorta have been conducted in the aortic arch or descending thoracic portion, where compromised reactivity is reported (Chung et al., 2007a,b,c, 2008; Yang et al., 2009, 2010a,b; Gibson et al., 2017).

Marfan syndrome (MFS) is equally prevalent in men and women (Mueller et al., 2013). Epidemiological evidence suggests that male patients with MFS have a higher risk of an aortic event (aortic surgery or aortic dissection) than females (Detaint et al., 2010; Franken et al., 2016; Groth et al., 2017). Nevertheless, the mechanisms that promote higher susceptibility to injury in males are not clear. Besides, pregnancy is associated with an increased risk of aortic dissection in MFS (Elkayam et al., 1995; Goland and Elkayam, 2009; Wanga et al., 2016). The pregnancy-induced myriad of aortic wall changes triggered by variations in hormone concentrations (Manalo-Estrella and Barker, 1967) and the altered hemodynamic load (Elkayam and Gleicher, 1998) could jointly render the aorta more susceptible to injury. However, it is unknown whether preexistent aortic alterations may predispose MFS females toward injury during pregnancy. Notably, no studies to date have directly attempted to examine the influence of sex on aortic disease in animal models of Marfan syndrome. These studies are fundamental to comprehend the complex pathophysiological mechanisms underlying aortic pathology in Marfan syndrome.

The efficacy and safety of pharmacological treatments in MFS is under investigation. An effective approach is the prophylactic surgical repair of the dilated ascending aorta, which involves non-negligible risks. Therefore, it is mandatory to go from bedside to bench for improved understanding of MFS pathogenesis, to uncover new potential effective pharmacological strategies, particularity for the management of both aortic root and ascending aorta dilatation. To this aim, the present study sought to examine whether there are regional and sex differences in thoracic aorta reactivity of mice heterozygous for the *Fbn1* allele encoding a missense mutation (*Fbn1*^C1039G/+^); the most common class of mutation in Marfan syndrome. The *Fbn1*^C1039G/+^ mice develop progressive aortic dilatation starting at about 2 months of age, and although mice rarely die from aortic dissection, they recapitulate most of the aortic complications observed in human MFS (Judge et al., 2004; Lee et al., 2016).

## Methods

### Animals

*Fbn1*^C1039G/+^ (Marfan) male and female mice and their wild-type littermates were obtained from The Jackson Laboratory/Charles River (Lyon, France). All mice were housed according to institutional guidelines (constant room temperature at 22°C, 12 h: 12 h light-dark cycle, 60% humidity, and access to food and water *ad libitum*). Experiments were conducted under the guidelines established by Spanish legislation (RD 1201/2005). They were approved by the Ethics Committee of the University of Barcelona, and were carried out in accordance with the recommendations in European legislation (Directive 2010/63/EU).

### Tissue preparation

**Figure 1:**
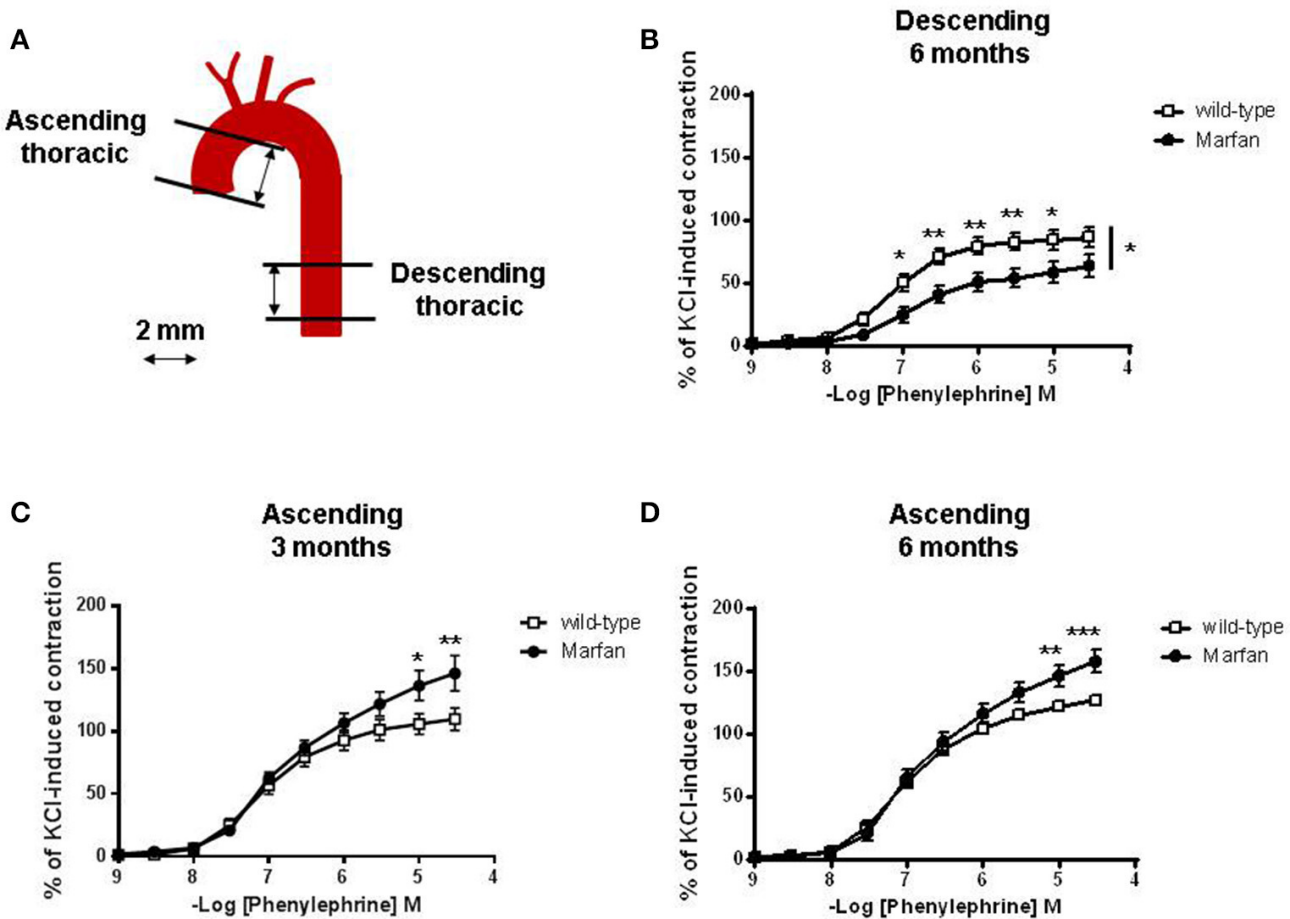
**(A)** Diagram illustrating the descending and ascending thoracic aorta segments used in the present study. Concentration-response curves to phenylephrine in descending **(B)** and ascending **(C,D)** aorta from wild-type and Marfan syndrome mice. Results are the mean ± SE from wild-type (descending *n* = 10; ascending *n* = 10) and Marfan syndrome (descending *n* = 10; ascending *n* = 10–13) mice. *P < 0.05, **P < 0.01, ***P < 0.001 by two-way ANOVA.

Segments of the ascending (vascular reactivity, mRNA levels, elastin breaks and protein levels) and descending (vascular reactivity) thoracic aorta were dissected (Figure 1A) free of fat and connective tissue in ice-cold physiological salt solution (PSS; composition in mM: NaCl 112.0; KCl 4.7; CaCl_2_ 2.5; KH_2_PO_4_ 1.1; MgSO_4_ 1.2; NaHCO_3_ 25.0 and glucose 11.1) gassed with 95% O_2_ and 5% CO_2_.

### Vascular reactivity

Three-mo-old wild-type (male *n* = 5; female *n* = 5) and Marfan (male *n* = 7; female *n* = 6) and 6-mo-old wild-type (male *n* = 11; female *n* = 11) and Marfan (male *n* = 12; female *n* = 10) mice were used. Segments (2 mm) of ascending and descending thoracic aorta were set up on an isometric wire myograph (model 410 A; Danish Myo Technology, Aarhus, Denmark) filled with PSS (37°C; 95% O_2_ and 5% CO_2_), as previously described (Onetti et al., 2013). The vessels were stretched to 6 mN, as described (Chung et al., 2007a), washed and allowed to equilibrate for 45 min. The tissues were contracted twice with 100 mM KCl every 5 min. After washing, vessels were left to equilibrate for a further 30 min before starting the experiments. Endothelial-dependent vasodilatations to acetylcholine (ACh; 10^−9^–10^−5^ M) were performed in 3 × 10^−6^ M phenylephrine (Phe)-precontracted vessels. Contractile responses mediated by α_1_-adrenoceptor stimulation were studied by evaluating Phe (10^−9^ to 3 × 10^−5^ M)-induced contraction. The effects of the non-selective nitric oxide synthase (NOS) inhibitor Nω-nitro-l-arginine methyl ester (L-NAME; 3 × 10^−4^ M), the non-selective cyclooxygenase (COX) inhibitor indomethacin (10^−5^ M), the intermediate (IK_Ca_) and large (BK_Ca_)-conductance calcium-activated potassium channel blocker charybdotoxin (100 nM), and the small-conductance K_Ca_ (SK_Ca_) channel blocker apamin (100 nM) were determined by adding each treatment 30 min before Phe- or ACh-induced responses.

### Quantitative real-time PCR

A different set of 6-mo-old wild-type (male *n* = 15; female *n* = 14) and Marfan (male *n* = 15; female *n* = 13) mice was used. mRNA expression was determined by quantitative real-time PCR (qRT-PCR) using SYBER green detection, as described (Novensa et al., 2010). Amounts of mRNA encoding COX-1, COX-2, eNOS, and iNOS were expressed relative to Ribosomal Protein S28 (Rps28), used as an internal control. qRT-PCR reactions were set up following the manufacturer's guidelines to SYBR green master mix (Thermo Fisher Scientific, Waltham, MA USA). Cycle threshold (Ct) values for each gene were referenced to the internal control [comparative Ct (ΔΔCt)] and converted to the linear form relative to corresponding levels in sex-matched wild-type levels (2^−ΔΔ*Ct*^). Primer sequences for murine genes used in this study are shown in Table 1.

**Table 1:** Primer sequences for quantitative real-time PCR.

**Gene (accession number)**	**Sequence (5′ → 3′)**
COX-1	*F: GAGCCGTGAGATGGGTGGGAGGG*
(NM_008969.3)	*R: TGGATGTGCAATGCCAACGGCT*
COX-2	*F: GTCAGGACTCTGCTCACGAAGGAAC*
(NM_011198.3)	*R: ACAGCTCGGAAGAGCATCGCAG*
eNOS	*F: TGTCACTATGGCAACCAGCGT*
(NM_008713.4)	*R: GCGCAATGTGAGTCCGAAAA*
iNOS	*F: TCAGCCACCTTGGTGAAGGGAC*
(NM_010927.3)	*R: TAGGCTACTCCGTGGAGTGAACA*
Rps28	*F: TAGGGTAACCAAAGTGCTGGGC*
(NM_016844)	*R: GACATTTCGGATGATAGAGCGG*

### Analysis of protein expression

Ascending aortic medial tissue from 6-mo-old wild-type (male *n* = 6; female *n* = 6) and Marfan (male *n* = 6; female *n* = 6) mice was homogenized using a bullet blender, ø 0.9–2 and 3 mm stainless steel beads (Next Advance, NY, USA) in 300 μl radioimmunoprecipitation assay buffer (RIPA, 10 mM Tris-HCl pH 7.5, 1 mM EDTA pH 7.5, 0.5 mM EGTA, 1% Triton X-100, 0.1% sodium desoxycholate, 0.1% SDS, 140 mM NaCl) and protease inhibitors. Protein concentrations were assessed using a colorimetric assay for protein concentration (Biorad, CA, USA). Twenty micrograms of protein per sample were analyzed by 10% (v/v) SDS-PAGE and transferred to nitrocellulose membranes. Primary antibodies to COX-1 (Santa Cruz, CA, USA), COX-2, eNOS, (p)eNOS (BD Transduction Laboratories, CA, USA), iNOS (Thermo Fisher, MA, USA) and actin (Sigma Aldrich, MO, USA) were incubated overnight in TBS 1% BSA. Protein bands were revealed using secondary IgG HRP conjugates (Promega, WI, USA) and Luminol Reagent together with Hyperfilm (Amersham Pharmacia Biotech, Uppsala, Sweden). Band intensities were measured by densitometry scanning using ImageJ software (National Institute of Health, Bethesda, MD, USA). Band intensities were relativized against actin as a loading control and the values of all groups were normalized for WT male average values.

### Analysis of elastin breaks

An additional set of 6-mo-old wild-type (male *n* = 8; female *n* = 7) and Marfan (male *n* = 8; female *n* = 8) mice was used. The ascending thoracic aorta of mice was dissected, rinsed and fixed in buffered formalin for 24 h, and posteriorly embedded in paraffin. Five-micron aortic sections were stained with Verhoeff-van Gienson (VVG) to visualize elastic fibers. Slides were examined using an Olympus BX60 microscope. Four representative VVG images of each mouse aorta were assessed and two blinded observers counted the number of elastin breaks.

### Statistics

All results are expressed as means ± SE of the number (*n*) of mice indicated in the figure legends. Relaxations to ACh are expressed as the percentage change from the Phe precontracted level. Contractions to Phe are expressed as a percentage of the tone generated by 100 mM KCl. The area under the curve was individually calculated from each concentration-response curve to Phe and was expressed as arbitrary units. Differences between concentration-response curves were assessed by two-way repeated measures ANOVA with Tukey's post-test. Differences between area under the curve, mRNA levels, elastin ruptures and protein expression were assessed by regular two-way ANOVA with Tukey's post-test. Data analysis was carried out using GraphPad Prism, Version 5 (GraphPad Software, La Jolla, CA). A value of *P* < 0.05 was considered significant.

## Results

### Influence of marfan syndrome on mice thoracic aorta reactivity

Contractile responses to KCl (100 mM) were unaffected by MFS in descending thoracic aortas (wild-type: 6.10 ± 0.87 mN, *n* = 10; Marfan: 6.23 ± 0.77 mN, *n* = 10) from 6-mo-old mice, and ascending aortas from 3-mo-old (wild-type: 5.43 ± 0.60 mN, *n* = 10; Marfan: 4.91 ± 0.41 mN, *n* = 13) and 6-mo-old (wild-type: 5.09 ± 0.25 mN, *n* = 22; Marfan: 4.98 ± 0.31 mN, *n* = 22) mice. The magnitude of the concentration-dependent contractions evoked by Phe was higher in the ascending (Figures 1C,D) than in the descending (Figure 1B) aorta. Phe-induced contractions were higher in the ascending aorta of 3-(Figure 1C) and 6-(Figure 1D) mo-old mice and decreased (*P* < 0.05; Figure 1B) in the descending aorta of 6-mo-old MFS mice. Conversely, there were no differences in endothelium-dependent ACh-induced vasodilatation between wild-type and Marfan mice in both thoracic aorta segments (results not shown).

### Influence of sex on phe-mediated contractions of marfan thoracic aorta

**Figure 2:**
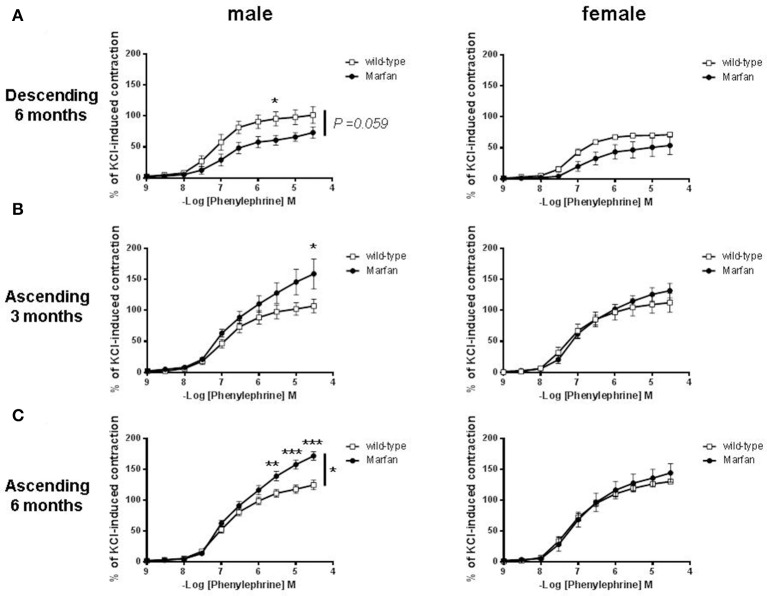
Concentration-response curves to phenylephrine in descending **(A)** and ascending **(B,C)** aorta from wild-type and Marfan syndrome male (left) and female (right) mice. Results are the mean ± SE from wild-type (male descending *n* = 5; male ascending *n* = 5; female descending *n* = 5; female ascending *n* = 5) and Marfan syndrome (male descending *n* = 5; male ascending *n* = 5–7; female descending *n* = 5; female ascending *n* = 5–6) mice. *P < 0.05, **P < 0.01, ***P < 0.001 by two-way ANOVA.

We then separated mice by sex to examine whether MFS affects the thoracic aorta reactivity of males and females differently. MFS did not alter contractile responses to KCl in descending aortas from 6-mo-old males (wild-type: 5.14 ± 0.96 mN, *n* = 5; Marfan: 5.31 ± 0.70 mN, *n* = 5) and females (wild-type: 7.05 ± 1.41 mN, *n* = 5; Marfan: 7.34 ± 1.40 mN, *n* = 5), or in ascending aortas from 3-mo-old males (wild-type: 4.16 ± 0.30 mN, *n* = 5; Marfan: 4.93 ± 0.61 mN, *n* = 7) and females (wild-type: 6.70 ± 0.85 mN, *n* = 5; Marfan: 4.88 ± 0.60 mN, *n* = 6), and ascending aortas from 6-mo-old males (wild-type: 4.92 ± 0.43 mN, *n* = 11; Marfan: 4.54 ± 0.42 mN, *n* = 12) and females (wild-type: 5.25 ± 0.27 mN, *n* = 11; Marfan: 5.50 ± 0.42 mN, *n* = 10). Contractions to Phe were similar in the wild-type mice from either sex (Figures 2A–C). The Marfan descending thoracic aorta from 6-mo-old mice showed a trend (*P* = 0.059) toward decreased contraction in males only (Figure 2A). Remarkably, at either age, Phe-induced contractions in ascending aortas from males were higher in MFS mice than in wild-type mice, whereas contractions in females were unaffected (Figures 2B,C).

### Influence of COX on sex differences in phe contractions of marfan ascending aorta

**Figure 3:**
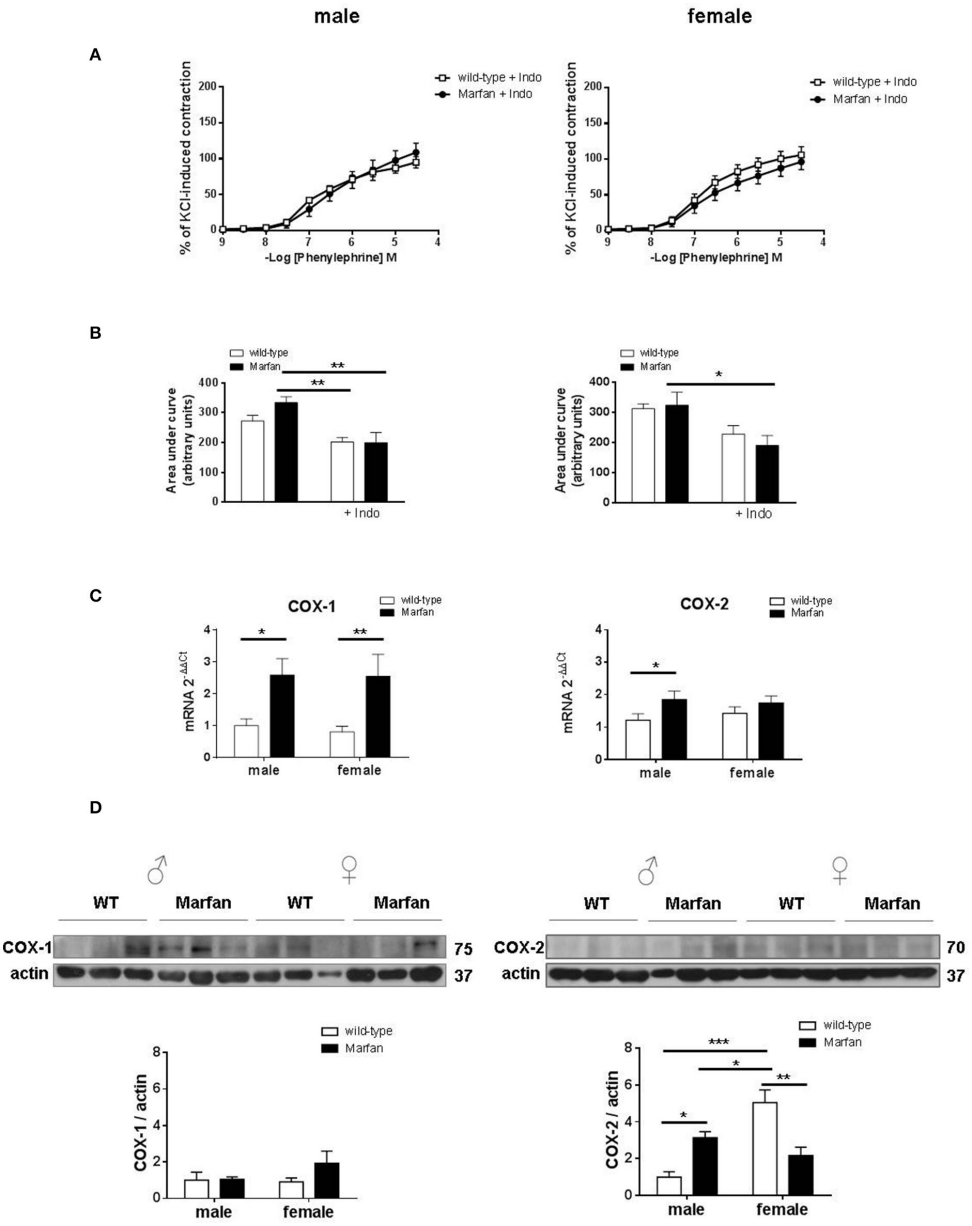
Concentration-response curves to phenylephrine **(A)** and analysis of area under the curve **(B)** in the presence of the non-selective cyclooxygenase (COX) inhibitor indomethacin (Indo; 10^−5^ M) in ascending aorta from wild-type and Marfan syndrome male (left) and female (right) mice. Results are the mean ± SE from wild-type (male *n* = 5; female *n* = 6) and Marfan syndrome (male *n* = 5; female *n* = 5) mice. **(C)** Comparative analysis of ascending aorta mRNA levels of COX isoforms, COX-1 and COX-2. mRNA levels are expressed as 2^−ΔΔ*Ct*^ using Ribosomal Protein S28 as internal control. Results are the mean ± SE from wild-type (male *n* = 13-15; female *n* = 14) and Marfan syndrome (male *n* = 13; female *n* = 12) mice. **(D)** Western blot analysis for COX-1 (left) and COX-2 (right) protein expression. Bar graphs (bottom) show the results of densitometric analyses from pooled data. The molecular weight (kDa) of the protein is shown on the right side of the blot. Results are the mean ± SE from wild-type (male *n* = 6; female *n* = 5–6) and Marfan syndrome (male *n* = 6; female *n* = 6) mice. *P < 0.05, **P < 0.01, ***P < 0.001 by two-way ANOVA.

Considering that aneurysmal expansion in human MFS initiates at the aortic root and progresses into the ascending portion, we subsequently focused on the mechanisms mediating sex differences in Marfan ascending aorta contractions from 6-mo-old mice. Previous studies demonstrated that COX is involved in the reactivity alterations of the Marfan descending thoracic aorta (Chung et al., 2007c). In the Marfan ascending aorta, the non-selective COX inhibitor indomethacin (10^−5^ M) reduced (*P* < 0.05) Phe-induced contractions in mice from both sexes, revealing a physiological COX-derived contractile influence (Figures 2C, 3A). Interestingly, Phe-induced contractions after indomethacin addition were similar in Marfan and wild-type mice in either sex (Figures 3A,B). These results suggest that COX activation underlies the Marfan-induced contractile alterations in males. We then measured mRNA (Figure 3C) and protein (Figure 3D) levels of COX isoforms. Although MFS significantly increased COX-1 mRNA levels in either sex, protein expression was unaffected. In contrast, mRNA and protein levels of COX-2 were augmented (*P* < 0.05) in Marfan males, which suggests that COX-2 was involved in the observed contractile alterations. Note that COX-2 protein expression was higher in wild-type females than in males, and MFS females showed reduced COX-2 expression.

### Influence of NOS on sex differences in phe contractions of marfan ascending aorta

**Figure 4:**
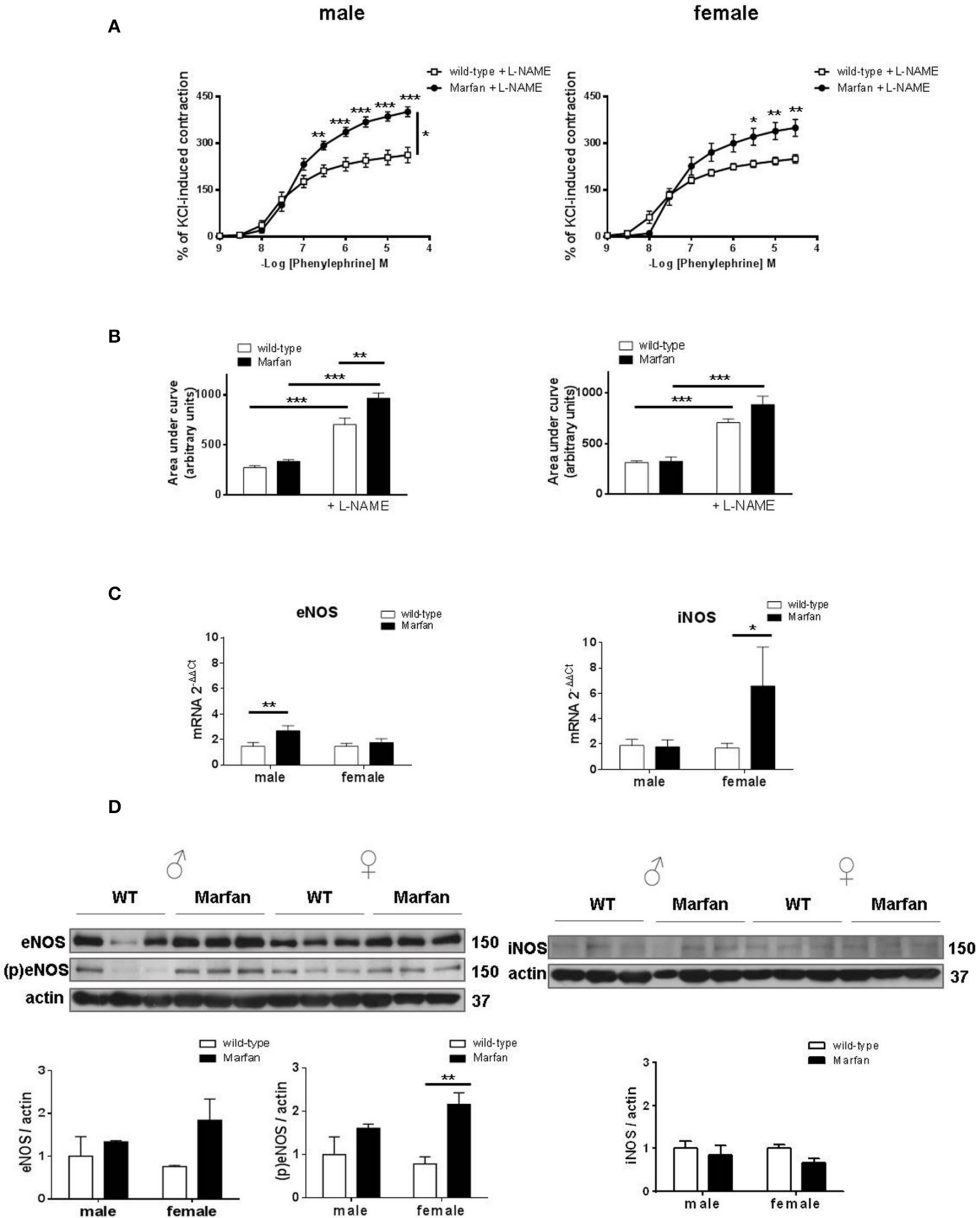
Concentration-response curves to phenylephrine **(A)** and analysis of area under the curve **(B)** in the presence of the nonselective nitric oxide synthase (NOS) inhibitor L-NAME (3 × 10^−4^ M) in ascending aorta from wild-type and Marfan syndrome male (left) and female (right) mice. Results are the mean ± SE from wild-type (male *n* = 4; female *n* = 5) and Marfan syndrome (male *n* = 5; female *n* = 6) mice. **(C)** Comparative analysis of ascending aorta mRNA levels of NOS isoforms, endothelial NOS (eNOS) and inducible NOS (iNOS). mRNA levels are expressed as 2^−ΔΔ*Ct*^ using Ribosomal Protein S28 as internal control. Results are the mean ± SE from wild-type (male *n* = 15; female *n* = 13–14) and Marfan syndrome (male *n* = 14–15; female *n* = 12–13) mice. **(D)** Western blot analysis for eNOS protein expression and phosphorylation at Ser-1177 (left), and iNOS protein expression (right). Bar graphs (bottom) show the results of densitometric analyses from pooled data. The molecular weight (kDa) of the protein is shown on the right side of the blot Results are the mean ± SE from wild-type (male *n* = 3–6; female *n* = 3–6) and Marfan syndrome (male *n* = 3–6; female *n* = 3–6) mice. *P < 0.05, **P < 0.01, ***P < 0.001 by two-way ANOVA.

Marfan syndrome (MFS) is associated with increased basal levels of NO (Yang et al., 2010b; Soto et al., 2016; Gibson et al., 2017; Oller et al., 2017). Therefore, we next evaluated the effects of the non-selective NOS inhibitor L-NAME (3 × 10^−4^ M) that similarly potentiated (*P* < 0.001) Phe-induced contractions in wild-type mice from both sexes (Figures 4A,B). The observed Marfan-induced increase in Phe-induced contractions (Figure 2C) in males was maintained after L-NAME addition (Figure 4A). However, in the presence of L-NAME, the area under the curve was greater (*P* < 0.01) in Marfan than in wild-type mice (Figure 4B). In Marfan females, L-NAME addition induced higher Phe-induced contractions than in the wild types (Figure 4A), though the increased area under the curve did not reach statistical significance (Figure 4B). These results suggest a greater negative influence of NO on Phe-induced contractions in either sex. Analysis of mRNA levels of NOS isoforms showed a significant Marfan syndrome-dependent increase in eNOS and iNOS in males and females, respectively (Figure 4C). However, eNOS phosphorylation was only significantly increased (*P* < 0.01) in Marfan females, whereas eNOS and iNOS protein expression remained unaltered in both groups (Figure 4D). These results suggest that changes in eNOS activation account, at least in part, for the increased NO negative influence on Phe-induced contractions in Marfan females, while this effect in Marfan males is not associated with changes in NOS expression.

### Influence of sex on ACh-mediated relaxations of marfan ascending aorta

**Figure 5:**
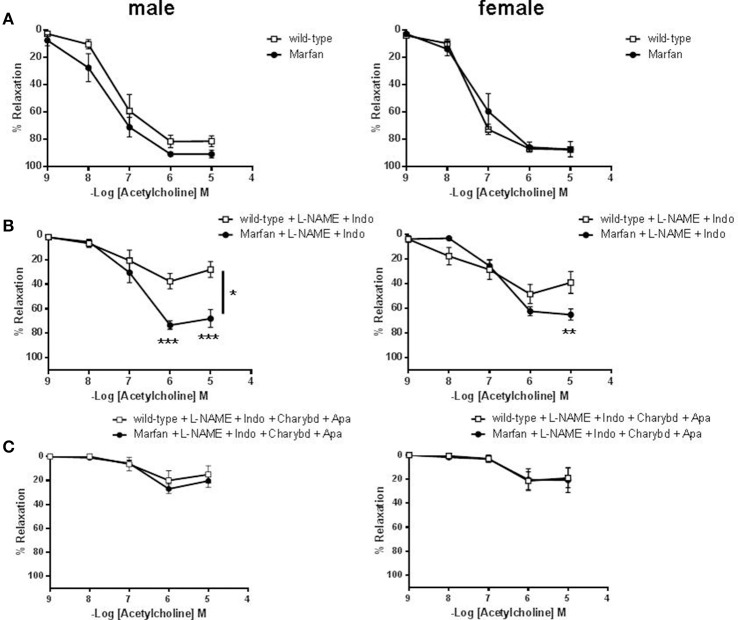
Concentration-response curves to acetylcholine in the absence **(A)** or presence of L-NAME (3 × 10^−4^ M) plus Indo (10^−5^ M) **(B)** or L-NAME plus Indo plus the IK_Ca_ and BK_Ca_ channel blocker charybdotoxin (Charybd; 100 nM) plus the specific SK_Ca_ channel blocker apamin (Apa; 100 nM) **(C)** in ascending aorta from wild-type and Marfan syndrome male (left) and female (right) mice. Results are the mean ± SE from wild-type (male *n* = 4–6; female *n* = 5–6) and Marfan syndrome (male *n* = 5–6; female *n* = 5–6) mice. *P < 0.05, **P < 0.01, ***P < 0.001 by two-way ANOVA.

Endothelium-dependent ACh-induced vasodilatation was similar in ascending aortas from 3- (results not shown) and 6-(Figure 5A) mo-old mice. In addition, indomethacin did not significantly modify ACh-mediated relaxation in either group (results not shown). However, incubation of L-NAME (results not shown) or L-NAME plus indomethacin (Figure 5B) to isolate endothelium-dependent hyperpolarization (EDH)-type dilation, showed higher aortic relaxations in Marfan than in wild-type. Subsequent addition of the IK_Ca_ and BK_Ca_ channel blocker charybdotoxin (100 nM) plus the specific SK_Ca_ channel blocker apamin (100 nM) almost abolished ACh-evoked EDH-type relaxations, and removed differences between Marfan and wild-type mice (Figure 5C).

### Influence of sex on ascending aorta elastin breaks in marfan syndrome

**Figure 6:**
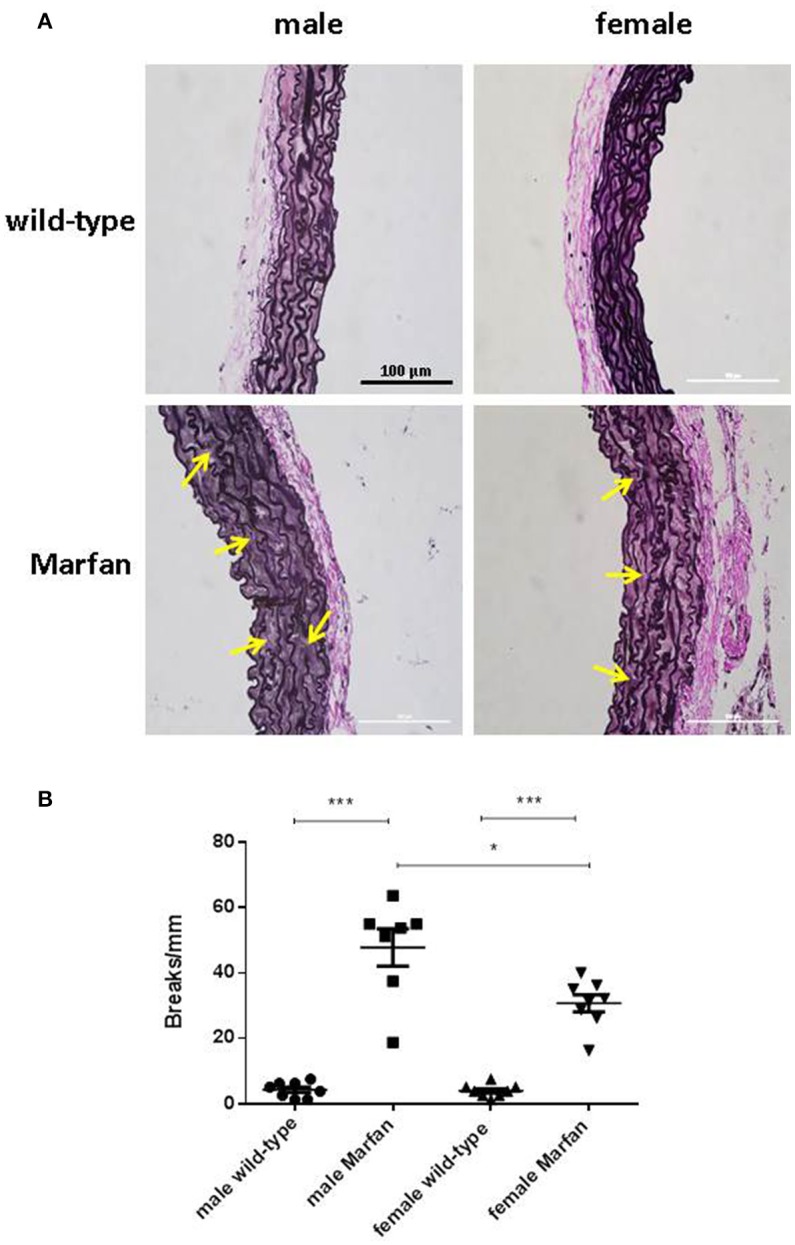
Representative images **(A)** and analysis of elastic fiber breaks **(B)** in the tunica media of the ascending aorta from wild-type and Marfan syndrome male and female mice. Scale bar represents 100 μm. Representative examples of elastin breaks are indicated with arrows. Results are the mean ± SE from wild-type (male *n* = 8; female *n* = 8) and Marfan syndrome (male *n* = 7; female *n* = 8) mice. *P < 0.05, ***P < 0.001 by two-way ANOVA.

Fragmentation of elastin is an important component of aneurysmal progression in Marfan syndrome. As expected, the number of elastin breaks was higher (*P* < 0.001) in the ascending aorta of MFS mice than wild-type mice (Figure 6). Furthermore, sex influenced the occurrence of elastin breaks in Marfan syndrome, since males showed a greater (*P* < 0.05) number of breaks than females.

## Discussion

There are two key novel findings in this study. Firstly, Marfan differentially affects ascending and descending portions of mice thoracic aorta, as evidenced by increased or decreased Phe-induced contractions, respectively. Secondly, sex differently affects ascending aorta reactivity in Marfan syndrome, since contractions only increased among males. Therefore, these results suggest the presence of regional and sex-related differences in ascending aorta reactivity, which is likely to be physiologically relevant in the management of thoracic aorta disease in Marfan syndrome.

A general paradigm of MFS pathophysiology is that aortic smooth muscle cells develop phenotypic alterations leading to aortic wall weakening (Chung et al., 2007b; Crosas-Molist et al., 2015). Previous studies reported that contractions of the aortic arch and descending thoracic aorta are decreased in *Fbn1*^C1039G/+^ mice (Chung et al., 2007a,b,c, 2008; Yang et al., 2009, 2010a,b; Gibson et al., 2017). However, in the current study, we demonstrate that MFS heterogeneously affects α_1_-adrenergic contractions to Phe in aneurismal (ascending aorta) and non-aneurysmal (descending aorta) tissue. Thus, whilst the descending portion showed impaired Phe-induced responses, we found that the ascending aorta, which is more prone to developing aneurysm in Marfan syndrome, showed augmented contractility. These results are consistent with those found in a previous study that indicates greater expression of contractile protein markers in human Marfan ascending aortas (Crosas-Molist et al., 2015). Our findings reveal for the first time the presence of regional differences in thoracic aorta reactivity in a mouse model of Marfan syndrome. The results are in agreement with mechanical abnormalities that have been found to be more pronounced in the ascending aorta than in the descending aorta in animal models of this pathology, which could render the ascending segment more susceptible to aneurysmal dilatation/rupture (Chung et al., 2007b; Bellini et al., 2016). The underlying determinants of the regional differences could be multiple, including differential mechanical loading that the pulsatile cardiac cycle transmits to the aortic tree (Prokop et al., 1970) and/or the different embryonic cell lineages of both aortic segments (Ruddy et al., 2013). For instance, a difference in α_1_-adrenergic receptor density, as reported in the canine thoracic aorta (Griendling et al., 1984) or in the population of α_1_-adrenergic receptor subtypes may create distinct environments in the ascending and descending aorta for the development of divergent functional alterations.

There is still controversy about whether MFS affects the thoracic aorta of men and women differently. However, epidemiological evidence suggests that Marfan males have a higher risk of aortic complications (Detaint et al., 2010; Franken et al., 2016; Groth et al., 2017). An important point is that the sex of MFS mice has only rarely been reported in previous studies of aortic reactivity. In the present study, we demonstrate that Marfan ascending aortas from males show a progressive increase in contractions from 3 to 6 months of age, whereas contractions in females are unaltered in the same time frame. These results provide the first evidence of sexual dimorphism in MFS thoracic aorta reactivity. A potential explanation for our results is that the MFS disease-causing mutation could have higher penetrance in male than female ascending aortas. To this end, we evaluated the influence of sex on elastin fragmentation as a measure of aortic disease progression. Although all Marfan groups had more elastic lamina breaks than the wild types, the breaks were more numerous in males than in females. These results suggest the presence of more pronounced aortic disease in 6-mo-old *Fbn1*^C1039G/+^ male mice, which correlates with the functional alterations that were observed. We cannot discard a protective effect by estrogens in female Marfan mice. In fact, this effect has been described in rat abdominal aortic aneurysms, where male predominance for aneurysm is also reported, and estradiol-treated rats exhibited smaller aneurysms (Ailawadi et al., 2004).

Aorta is a poorly innervated vessel that highly depends on circulating catecholamines to maintain sympathetic activity. We suggest that heightened α_1_-adrenergic receptor-dependent contractions of male Marfan ascending aortas might be an orchestrated response to excessive aortic enlargement. However, at this stage, it is unclear whether maintenance of these alterations may be beneficial in the long-term, as for instance:

(i) sympathetic overactivity in cardiovascular disorders (i.e. hypertension, myocardial infarction) is life threatening (Grassi, 2010); (ii) sympathetic tone is chronically elevated in human aging (Casey et al., 2012); and (iii) excessive aortic vasoconstriction increases cardiac afterload, which can be detrimental in MFS patients, particularly in the setting of congestive heart failure.

The principal vascular cell type involved in MFS pathogenesis is the smooth muscle cell (Crosas-Molist et al., 2015; Perrucci et al., 2017). However, abnormalities of endothelial function disrupt circulatory homeostasis, which could aggravate Marfan syndrome-induced vascular damage. Impaired endothelium-dependent vasodilation to ACh has been reported in Marfan descending thoracic aortas of 3- and 6-mo-old mice (Chung et al., 2007a). In the current study, relaxations to ACh were unaltered in the ascending aorta of 6-mo-old Marfan mice. Importantly, we noted that EDH-type relaxations were augmented in Marfan animals from both sexes. In large conduit arteries such as aorta, agonist-induced endothelium-dependent relaxations involve both NO and prostacyclin, whereas EDH-type relaxations are more potent in smaller vessels (Chataigneau et al., 1999; Brandes et al., 2000; Takaki et al., 2008). Increased EDH-type relaxations in microvessels could compensate for endothelial abnormalities in cardiovascular disease (Shimokawa and Urakami-Harasawa, 1999). Nevertheless, although much less studied, EDH-type relaxations may also serve as a backup mechanism for endothelial responses of mice thoracic aorta (Shen et al., 2003; Csányi et al., 2012). Therefore, we propose that EDH-dependent preservation of endothelium function in MFS ascending aorta could be a plausible adaptive mechanism to maintain endothelium-dependent relaxation.

Previous studies reported that impaired Phe-induced contractions of the Marfan descending thoracic aorta are associated with a shift toward reduced expression of COX-1 and enhanced expression of COX-2 (Chung et al., 2007c). Notably, a partly different scenario occurs in the Marfan ascending aorta, in which COX-dependent increases in Phe-induced contractions in males are coupled to exclusive upregulation of COX-2 expression. In contrast, COX-2 expression was reduced in Marfan females, an effect that was not accompanied by changes in Phe-induced contractions. Therefore, we could speculate that increased COX-2-derived contractile prostanoids (Álvarez et al., 2005) may participate, at least partly, in enlarged Phe contractions in males. Besides, elevated COX-2 expression is associated with increased metalloproteinase activation (Tsujii et al., 1997), which in turn is associated with aortic elastic fiber fragmentation and degradation in MFS (Chung et al., 2007b). Consistently, increased COX-2 expression correlated with a larger number of elastin breaks in the ascending aorta of Marfan males than in Marfan females. The underlying driving force for increased COX-2 expression is unclear, but it could be linked to loss of vessel elasticity and increase in pulse wave velocity of the Marfan thoracic aorta (Chung et al., 2007c). There is accumulating evidence connecting excessive basal levels of NO and Marfan syndrome-induced aortic pathology (Yang et al., 2010b; Soto et al., 2016; Gibson et al., 2017; Oller et al., 2017). Consistently, our results reveal increased participation of NO as a negative modulator of Phe-induced contractions in Marfan mice of either sex. Moreover, our findings provide evidence of the potential source of altered NO generation in female Marfan ascending aortas, where eNOS phosphorylation was upregulated. These results are consistent with the protective effects of estrogen on vascular function, which have been largely associated with upregulated eNOS (Chambliss and Shaul, 2002). Interestingly, a recent study has shown that dysregulated NO production via iNOS plays an important role in MFS aortic dilatation (Oller et al., 2017). Therefore, the participation of an iNOS-derived NO pool in Marfan mice of either sex caused by increases in enzyme activity should not be excluded.

Altogether, the results of the present study provide evidence of regional and sex-related differences in thoracic aorta reactivity in a representative model of Marfan syndrome. Increased α-adrenergic contractions may render the ascending aorta of males more sensitive to circulating catecholamines, which might be a short-term adaptive functional response against excessive aortic enlargement. Nevertheless, further studies are necessary to verify whether these alterations can contribute to increased thoracic aorta vulnerability in male MFS patients. The present study suggests that the development of an effective drug treatment against excessive aortic enlargement should consider these functional divergences, which might open the door to safer and more effective personalized therapy in Marfan syndrome.

## Author contributions

FJ-A: conceived the study, designed and executed the experiments, guided the experimental design, analyzed and interpreted the data, wrote and revised the manuscript. GE and EV: guided the experimental design, data analysis and interpretation, read and revised the manuscript. AD, A-MS, FB, TM, and LB: executed the experiments, data analysis and interpretation, read and revised the manuscript. All authors gave final approval of the manuscript to be published.

### Conflict of interest statement

The authors declare that the research was conducted in the absence of any commercial or financial relationships that could be construed as a potential conflict of interest. The reviewer LAML and handling Editor declared their shared affiliation.
